# The Prognostic Value of Baseline Lymphocyte, Neutrophil, and Monocyte Counts in Locally Advanced Cervical Carcinoma Treated with Radiation

**DOI:** 10.1155/2017/8584605

**Published:** 2017-01-23

**Authors:** Sareena Singh, Justin Himler, Christa I. Nagel, Kimberly Resnick

**Affiliations:** ^1^Division of Gynecologic Oncology, MetroHealth Medical Center, Cleveland, OH, USA; ^2^Department of Obstetrics and Gynecology, MetroHealth Medical Center, Cleveland, OH, USA; ^3^Division of Gynecologic Oncology, University Hospitals Case Medical Center, Cleveland, OH, USA

## Abstract

*Background*. To determine the prognostic significance of pretreatment levels of circulating lymphocyte (CLC), neutrophil (CNC), and monocyte (CMC) counts in patients with locally advanced cervical carcinoma (CC) treated with definitive radiation.* Methods*. A retrospective, dual-institution review of patients with Stage IB2-IVA CC from 2005 to 2015. Progression-free (PFS) and Overall Survival (OS) were determined for high and low CLC, CNC, and CMC groups. Multivariate analysis was used to confirm prognostic value of baseline leukocyte counts.* Results*. 181 patients were included. Median follow-up time was 26 (3–89) months. CNC had no effect on PFS or OS. PFS was similar between CMC groups; however, OS was significantly improved for patients with low CMC (62.5 versus 45.3 months, *p* = 0.016). High CLC was associated with improved PFS (48.5 versus 27.8 months, *p* = 0.048) and OS (58.4 versus 34.9 months, *p* = 0.048). On multivariate analysis, high CNC was associated with increased relapse risk (HR 1.12, *p* = 0.006) and low CLC was associated with increased mortality risk (HR 0.67, *p* = 0.027).* Conclusion*. This study demonstrates that leukocyte values can provide prognostic information in CC. These hypothesis-generating findings warrant further prospective investigations.

## 1. Introduction

Leukocytes, as a whole, play an integral role in the innate immune response of a host against damage to tissue, infection, and neoplasia. It has been proposed that the roles of circulating lymphocytes, monocytes, and neutrophils may differ in cancer patients when compared to patients without a diagnosis of malignancy. Studies have also shown an association between the number of innate immune cells and the prognosis of a variety of different cancers. It has been hypothesized that the interaction of the leukocytes with the cancer microenvironment may affect metastatic changes in the disease process, which could then affect prognosis [1].

In cervical cancer, it has been demonstrated that higher circulating neutrophil counts (CNCs) are present when compared to controls [2]. The prognostic significance of higher levels of CNCs, however, has yet to be elucidated in this population. Decreased pretreatment circulating lymphocyte counts (CLCs) have been associated with decreased survival in cervical cancer patients [3]. This association between increased CLC and improved survival has also been demonstrated in other malignancies, such as laryngeal cancer and colorectal cancer, as well [4–6]. In cancer of the oral cavity, higher circulating monocyte counts (CMCs) prior to treatment are associated with poor outcomes [7]. CMCs have also been demonstrated to have prognostic indication in cancers of the stomach, head and neck, melanoma, liver, and colorectum [8]. The association between CMCs and outcome in cervical cancer, however, has only been investigated in one study, which also showed that higher levels of CMCs are associated with poorer oncologic outcomes [8].

The role that the immune system plays in cancer development, progression, and recurrence is currently the subject of multiple investigations. Since cervical cancer is one of several human malignancies that has been identified to develop as a direct effect of infection by a virus (in this case, Human Papilloma Virus), the effect of a host's immune response against malignant progression becomes especially relevant. The objective of this study was to further investigate the prognostic significance of pretreatment levels of circulating lymphocytes, monocytes, and neutrophils on outcomes of patients with cervical cancer treated with radiation.

## 2. Materials and Methods

An IRB approved retrospective chart review was performed of all patients with newly diagnosed cervical cancer at University Hospitals Case Medical Center (Cleveland, OH) and MetroHealth Medical Center (Cleveland, OH) from 2005 to 2015. Patients who completed definitive radiation (with or without sensitizing chemotherapy) for locally advanced (FIGO Stages IB2 through IVA) were included. We did not include patients who underwent hysterectomy as part of initial treatment. Demographic, clinical, pathologic, treatment, and follow-up information was abstracted from medical records. Patients who did not complete radiation or progressed while on initial treatment were excluded. Patients who did not have available pretreatment laboratory data also were also excluded. Pretreatment CLC, CMC, and CNC values were obtained from complete blood counts with differentials that were performed within 30 days of initiation radiation therapy. Median values of CLCs, CMCs, and CNCs were used to dichotomize patients into high versus low groups. Clinical characteristics and outcomes were compared between patients with high and low circulating leukocyte levels. Progression-free survival (PFS) was calculated from date of diagnosis to date of first documented recurrence (as determined on imaging or physical examination). Overall survival (OS) was calculated from date of diagnosis to date of last follow-up.

All statistical analyses were performed with SPSS v.22.0 (IBM, Chicago, IL). Associations between categorical variables were determined using chi-squared and Fisher exact tests. Differences between means of continuous variables were determined using Student's *t*-tests. OS and PFS were calculated using the Kaplan-Meier method with a log-rank test for statistical significance. Univariate and multivariate analyses using Cox Proportional Hazard Regression were used to identify predictors of mortality risk and relapse risk. All demographic and clinical variables listed in Table 1 were included in these analyses, as these have previously all been established as risk factors for PFS and OS in cervical cancer. An *α* level of 0.05 was utilized to determine statistical significance.

## 3. Results

**Figure 1:**
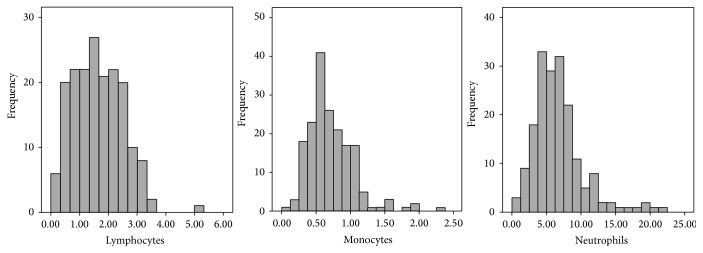
Histograms depicting pretreatment lymphocyte, monocyte, and neutrophil counts.

A total of 181 patients with a diagnosis of locally advanced cervical cancer were identified from 2005 to 2015. The median follow-up time for these patients was 26 (3–89) months. The demographic and clinical characteristics of the entire cohort are shown in Table 1. Median age was 52 years old. Most patients were Caucasian and had tumors of squamous histology. Ninety-three percent of patients received sensitizing chemotherapy concurrently with radiation. The majority of patients had Stage II or Stage III disease. The CLC, CMC, and CNC distributions for all patients are depicted in Figure 1. The median CLC was 1.60 (0.15–5.10), the median CMC was 0.66 (0.12–2.27), and the median CNC was 6.2 (0.74–22.48).  Table 2 shows demographic and clinical characteristics of patients as divided by into groups based on high versus low pretreatment circulating leukocyte count.

### 3.1. Relation of Circulating Neutrophil Counts on Cervical Cancer Outcomes

**Figure 2:**
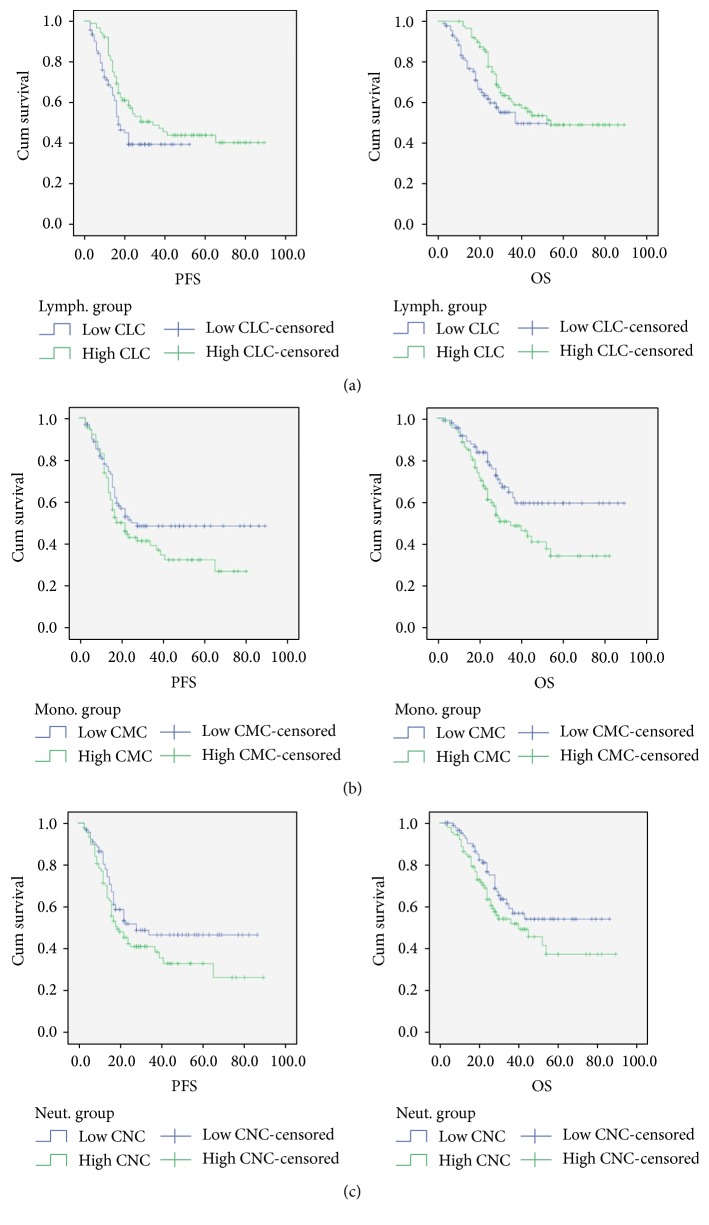
Kaplan-Meier survival curves for PFS and OS in months based on Pretreatment high versus low CLC (a), CMC (b), and CNC (c) groups.

Table 2 compares basic clinical and demographic characteristics of patients with high CNC versus low CNC. Patients with high CNC counts had significantly lower BMIs and were significantly more likely to have squamous histology. There were no significant differences between age, race, smoking status, stage, and use of concurrent chemotherapy. At the end of the data collection period, 40.4% of patients in the high CNC group experienced a cancer recurrence, while 53.3% of patients in the low CNC group had a recurrence. Progression-free survival did not differ significantly between the 2 groups (Figure 2). Patients in the high CNC group had a PFS of 38.7 months (95% CI 30.5–46.8) and patients in the low CNC groups had a PFS of 48.3 months (95% CI 40.4–56.2) (*p* = 0.102). There were 41 (53.9%) deaths in the high CNC group and 30 (67.4%) deaths in the low CNC group. Overall survival also did not differ significantly between the 2 groups: 49.5 months (95% CI 41.1–57.9) in the high CNC group versus 57.8 months (95% CI 50.1–65.6) (*p* = 0.093) (Figure 2). In the univariate analysis, a higher CNC was associated with a significantly increased relapse risk (HR 1.12, *p* < 0.001) and mortality risk (HR 1.11, *p* < 0.001) (Table 3). In the multivariate analysis, however, a higher CNC was significantly associated with a higher relapse risk (HR 1.12, *p* = 0.006) but not significantly associated with mortality risk (Table 4).

### 3.2. Relation of Circulating Monocyte Counts on Cervical Cancer Outcomes

Table 2 compares basic clinical and demographic characteristics of patients with high CMC versus low CMC. Clinical and demographic factors did not differ significantly between patients in the high CMC group versus those in the low CMC group, except for smoking status. There were significantly more smokers in the high CMC group (61.8% versus 44.6%, *p* = 0.025). In the high CMC group, 39.3% of patients experienced a disease recurrence, while 54.3% patients in the low CMC group had a disease recurrence. PFS did not differ significantly between the high CMC group (36.6 months, 95% CI 29.7–43.5) and the low CMC group (50.4 months, 95% CI 42.1–58.7) (*p* = 0.133) (Figure 2). There were 45 (49.4%) deaths in the high CMC group and 26 (28.3%) deaths in the low CMC group. Overall survival was significantly higher for patients in the low CMC group (62.5 months, 95% CI 54.2–70.7) as compared to the high CMC group (45.3 months, 95% CI 38.2–52.3) (*p* = 0.016) (Figure 2). In the univariate analysis, a higher CMC was significantly associated with a higher mortality risk (HR 2.45, *p* = 0.002) but not significantly with higher relapse risk (Table 3). On multivariate analysis, however, a higher CMC was nonsignificant (Table 4).

### 3.3. Relation of Circulating Lymphocyte Counts on Cervical Cancer Outcomes

When basic clinical and demographic characteristics of patients with high CLC versus low CLC were compared, the only factor noted to be significantly different between the 2 groups was smoking status (Table 2). There were more smokers in the high CLC group than in the low CLC group. There were 47 (47.2%) cases of cancer recurrence in the high CLC group and 49 (46.7%) cases of cancer recurrence in the low CLC group. PFS differed significantly between the 2 groups (Figure 2). PFS was 48.5 months (95% CI 40.8–56.3) for the high CLC group compared to 27.8 months (95% CI 23.4–32.2) for the low CLC group (*p* = 0.048) (Figure 2). Thirty-eight (57.3%) deaths were observed in the high CLC group and 33 (64.1%) deaths were observed in the low CLC group. Overall survival also differed significantly between the 2 groups. OS was 58.4 months (95% CI 51.3–65.5) for the high CLC group and was 34.9 months (95% CI 30.5–39.4) for the low CLC group (*p* = 0.048) (Figure 2). While on univariate analysis, there was no significant association between CLC and relapse or mortality risk (Table 3), lower CLC was associated with a significant increase in mortality risk on multivariate analysis (HR 0.67, *p* = 0.027) (Table 4).

## 4. Discussion

A variety of studies have examined the associations between pretreatment circulating leukocytes and oncologic outcomes; however, this is the first study to concurrently survey the associations between 3 different leukocyte measures and outcomes in locally advanced cervical carcinoma. Our study showed possible associations between all 3 leukocyte measures and outcomes: higher pretreatment levels of neutrophils and monocytes are associated with poorer outcomes, while higher pretreatment levels of lymphocytes are associated with improved outcomes. These findings, and the findings from previous studies, emphasize the important role the immune system plays in cancer outcomes.

Neutrophils are the most abundant type of white blood cell in our circulatory system and contribute early to a host's response to both inflammation and infection [9]. Inflammation itself has been documented to play a fundamental role in cancer development, specifically with regard to growth and metastasis of tumors [9, 10]. It has been demonstrated that neutrophils are recruited from the bloodstream and into tumors where tumor-associated neutrophils (TAN) contribute to cancer initiation, angiogenesis, invasion, progression, and dissemination [11]. In cervical cancer patients, TANs (which are derived from circulating blood neutrophils) can be found in tumor nests, peritumoral tissues, and the cervical stroma [12]. The highest concentration of neutrophils is in the peritumoral tissue. In an immunohistochemistry study of whole tissue sections, Carus et al. studied the association between tumor-associated neutrophils and cervical cancer [11]. This group found that high levels of peritumoral neutrophils were an independent prognostic factor for shorter progression-free survival in localized cervical cancer. Our results did not show a difference in PFS or OS between patients in the low CNC group and the high CNC group. In the univariate analysis, increased CNCs were associated with an increased relapse risk and mortality risk; however, on multivariate analysis, increased CNCs were only associated with increased relapse risk. Our results were in concordance with the results from the Carus study. Perhaps a more robust patient sample would have demonstrated a difference in PFS and OS between the 2 groups in our study. Additionally, PTNs are derived from circulating neutrophils and it is possible that circulating levels of neutrophils may not accurately reflect the extent of TANs.

The relationship between circulating monocyte counts and cancer prognosis involves the activation of the innate immune response [13]. In this system, antigen-presenting cells (APCs) display antigen complexes that present major histocompatibility complex on the cell surface [8, 14]. Receptors on T-Cells are then used to help T-Cells identify and recognize this complex. APCs are derived from peripheral blood monocytes and have been demonstrated to have a role in the host's immune response to tumors, including initiation, programming, and regulation [15–17]. The presence of an increased number of cells derived from monocytes can create a state of low-grade inflammation via secretion of a variety of cytokines [18]. Several of these cytokines, including TNF-*α*, IL-1, and IL-6, have themselves been shown to have an association with worse prognosis in patients with cancer [7, 19, 20]. Moreover, other studies have demonstrated that increased levels of peripheral monocytes may be associated with higher levels of marrow-derived myelomonocytic cells [21]. These cells function to stabilize tumor vasculature by infiltrating tumor masses and differentiating into tumor-associated macrophages, which then release a multitude of angiogenic factors and cytokines, such as vascular endothelial growth factor (VEGF), TNF-*α*, and matrix metalloproteinase-9 [22, 23]. The role of angiogenesis as a negative prognostic factor in cervical cancer has been well studied and well documented [24–26]. More recently, in a study by the Gynecologic Oncology Group (Protocol 24), it was demonstrated that addition of bevacizumab (a monoclonal antibody directed against VEGF) to systemic chemotherapy resulted in improved overall survival and higher treatment response rates [27].

In a retrospective study of nearly 800 patients, Lee et al. examined the prognostic value of pretreatment circulating monocyte counts in patients with cervical cancer [8]. Patients with Stage IB1 to IVA were included. Treatment was dependent of physician preference and included surgery, radiation, chemotherapy, or a combination of more than one modality. The majority (58.8%) of patients had stage IB1 disease and most patients (38.5%) underwent radical hysterectomy alone as primary therapy. Pretreatment monocyte count was compared with SCC-Ag levels to determine the ability of these levels to be markers for clinical outcome. In a multivariable analysis, the authors found that pretreatment CMC was an independent prognostic factor for PFS and OS when patients with locally advanced disease (Stage IIB–IVA, *n* = 202) were subselected from the entire cohort. The results of our study corroborate with those above. We found that when patients were dichotomized into low CMC versus high CMC groups, patients in the low CMC group had significantly improved overall survivals (no difference in PFS). In univariate analysis, a high CMC value was associated with a statistically significant increase in mortality risk, although this association did not persist through the multivariable analysis. Taking into account the results from Lee et al. and our work, it would be reasonable to consider using pretreatment monocyte counts as a possible factor to group patients with locally advanced disease based on OS.

The role of lymphocytes and lymphocytopenia in cancer has been well studied. Lymphopenia is associated with poorer survival and many types of human malignancies, including cancers of the cervix, larynx, colorectum, and oropharynx [3–6]. Previous studies have shown the presence of tumor infiltrating lymphocytes (TILs) in many different tumor types, further strengthening the concept that malignancy activates the host immune system [28]. The presence of TILs in tumor tissue is associated with survival benefits, as TILs work to delay tumor progression through a variety of mechanisms [29–32]. A number of studies have shown that, specifically in cervical cancer, lower values of baseline lymphocytes are associated with shorter PFS [33–35]. The results from our study are concordant with these findings. We also demonstrated that higher CLC were associated with improved PFS and OS. In multivariate analysis, higher CLC were significantly associated with increased mortality risk with a nonsignificant association with relapse risk. In a recent article by Wu et al., the presence of posttreatment lymphocytopenia in cervical cancer patients was examined [3]. These authors found that severe and prolonged lymphopenia was observed in half of the patients included in their retrospective study. Although their results did not reach statistical significance, their findings suggested that posttreatment lymphopenia may also be associated with decreased survival.

## 5. Conclusion

In conclusion, we present data to potentially support the use of pretreatment baseline leukocyte values as predictive markers for oncologic outcome in patients with locally advanced cervical cancer treated with primary radiotherapy with or without sensitizing chemotherapy. Limitations of this study are inherent to its small sample size and retrospective nature. The strongest association between pretreatment leukocyte levels and outcomes was seen with lymphocytes, although associations between pretreatment levels of monocytes and neutrophils and cancer outcomes are also suggested. These measurements could potentially be used to guide treatment decisions and to help predict prognosis in patients with cervical cancer and warrant further study.

## 6. Future Directions

While the field of cancer immunotherapy is rapidly expanding, there is much that we do not know about predicting response to therapies including vaccines, monoclonal antibodies, and cytokine treatments. Cervical cancer, a malignancy caused by direct viral infection, seems a logical malignancy to focus on further. Vaccination therapy, such as ADXS11-001, is being touted as an active immune therapy in squamous cell cancer of the cervix [36]. The prediction of patient response to immunotherapies is a question that still needs to be answered. For example, in renal cell patients being treated with IL-2, pretreatment lymphocyte counts were predictive of improved overall survival independent of tumor response and patient characteristics [37]. Additionally, pretreatment neutrophil and total leukocyte count were predictive of shorter overall survival in melanoma patients undergoing IL-2 treatment [38]. There is some scientific data to support the notion that peripheral immune cells are suitable surrogates for tumor infiltrating immune cells [39, 40]. The data regarding predictions of response to vaccines using peripheral blood samples is scarce. Future directions of our work may include prospective studies with peripheral blood samples and flow cytometry in this patient population or additional translational studies using banked blood from patients who have undergone vaccine therapy.

## Figures and Tables

**Table 1:** Demographic and clinical characteristics of the entire cohort.

Clinical characteristic	*n* = 181
Median age (range) (yrs)	52 (25–92)
Race	
Caucasian	121 (67)
African-American	53 (29)
Other	7 (4)
Median BMI (range) (k/m^2^)	27.1 (15.2–67.7)
Smoker	
Yes	96 (53)
No	85 (47)
Histology	
Squamous	160 (88.4)
Adenocarcinoma	20 (11)
Adenosquamous	1 (0.6)
Stage	
IB2	32 (17.7)
IIA	22 (12.2)
IIB	41 (22.7)
IIIA	9 (5)
IIIB	61 (33.7)
IVA	16 (8.8)
Treatment	
RT alone	12 (6.6)
CRT	169 (93.4)

**Table 2:** Clinical and demographic characteristics of patients based on high versus low pretreatment leukocyte counts.

Clinical characteristic	High CNC (*n* = 89)	Low CNC (*n* = 92)	*p*	High CLC (*n* = 89)	Low CLC (*n* = 92)	*p*	High CMC (*n* = 89)	Low CMC (*n* = 92)	*p*
Mean age (yrs)	53.2 ± 13.4	54.4 ± 14.0	0.56	52.1 ± 14.0	55.4 ± 13.2	0.12	53.9 ± 14.1	53.6 ± 13.3	0.89
Race			0.79			0.06			0.27
Caucasian	58 (65.2)	63 (68.5)		52 (58.4)	69 (75)		55 (61.8)	66 (71.7)	
AA	28 (31.5)	25 (27.2)		33 (37.1)	20 (21.7)		31 (34.8)	22 (23.9)	
Other	3 (3.4)	4 (4.3)		4 (4.5)	3 (3.3)		3 (3.4)	4 (4.3)	
Mean BMI (kg/m^2^)	27.0 ± 8.2	30.2 ± 7.8	**0.008**	28.4 ± 6.8	28.8 ± 9.3	0.75	27.6 ± 7.2	29.6 ± 9.0	0.11
Smoker			0.66			**0.025**			**0.025**
Yes	49 (55.1)	47 (51.1)		55 (61.8)	41 (44.6)		55 (61.8)	41 (44.6)	
No	40 (44.9)	45 (48.9)		34 (38.2)	51 (55.4)		34 (38.2)	51 (55.4)	
Histology			**0.046**			0.50			0.24
Squamous	83 (93.3)	77 (83.7)		77 (86.5)	83 (90.2)		82 (92.1)	78 (84.8)	
Adenocarcinoma	5 (5.6)	15 (16.3)		11 (12.4)	9 (9.8)		7 (7.9)	13 (14.1)	
Adenosquamous	1 (1.1)	—		1 (1.1)	—		—	1 (1.1)	
Stage			0.61			0.10			0.99
IB2	14 (15.7)	18 (19.6)		14 (15.7)	18 (19.6)		15 (16.9)	17 (18.5)	
IIA	10 (11.2)	12 (13)		9 (10.1)	13 (14.1)		11 (12.4)	11 (12)	
IIB	22 (24.7)	19 (20.7)		28 (31.5)	13 (14.1)		20 (22.5)	21 (22.8)	
IIIA	5 (5.6)	4 (4.3)		4 (4.5)	5 (5.4)		4 (4.5)	5 (5.4)	
IIIB	33 (37.1)	28 (30.4)		29 (32.6)	32 (34.8)		31 (34.8)	30 (32.6)	
IVA	5 (5.6)	11 (12)		5 (5.6)	11 (12)		8 (9)	8 (8.7)	
Treatment			0.24			0.59			0.56
RT alone	8 (9)	4 (4.3)		6 (6.7)	6 (6.5)		7 (7.9)	5 (5.4)	
CRT	81 (91)	88 (95.7)		83 (93.3)	86 (93.5)		82 (92.1)	87 (94.6)	

**Table 3:** Univariate analysis of potential factors associated with OS and PFS.

Variable	Overall survival		Progression-free survival	
	Hazard ratio [95% CI]	*p*	Hazard ratio [95% CI]	*p*
Age (years)	1.00 [0.99–1.02]	0.611	1.01 [0.99–1.02]	0.376
Race				
White	Reference		Reference	
Black	0.79 [0.19–3.29]	0.749	0.94 [0.30–3.01]	0.920
Other	0.93 [0.22–3.97]	0.927	1.07 [0.33–3.5]	0.912
BMI (kg/m^2^)	0.98 [0.94–1.01]	0.149	0.99 [0.96–1.01]	0.31
Smoker				
Smoker	Reference		Reference	
Nonsmoker	1.17 [0.74–1.88]	0.503	1.04 [0.70–1.56]	0.851
Histology				
Squamous	Reference		Reference	
Adenocarcinoma	0.37 [0.05–2.71]	0.330	0.57 [0.80–4.14]	0.582
Adenosquamous	0.42 [0.05–3.38]	0.418	0.80 [0.10–6.11]	0.827
Stage				
IB2	Reference		Reference	
IIA	0.31 [0.10–0.91]	**0.034**	0.35 [0.15–0.78]	0.10
IIB	0.31 [0.09–1.05]	0.061	0.32 [0.13–0.79]	0.14
IIIA	0.50 [0.20–1.25]	0.139	0.35 [0.16–0.73]	**0.006**
IIIB	1.39 [0.46–4.17]	0.561	0.47 [0.15–1.48]	0.197
IVA	1.14 [0.52–2.57]	0.761	0.85 [0.45–1.63]	0.631
Treatment				
RT alone	Reference		Reference	
CRT	2.33 [1.06–5.12]	**0.036**	2.45 [1.26–4.74]	**0.008**
Circulating neutrophil count	1.11 [1.05–1.17]	**<0.001**	1.12 [1.03–1.19]	**<0.001**
Circulating lymphocyte count	0.80 [0.60–1.06]	0.119	0.83 [0.65–1.07]	0.147
Circulating monocyte count	2.45 [1.39–4.31]	**0.002**	2.15 [1.23–3.75]	**0.007**

**Table 4:** Multivariate analysis of potential factors associated with OS and PFS.

Variable	Overall survival		Progression-free survival	
	Hazard ratio [95% CI]	*p*	Hazard ratio [95% CI]	*p*
Age (years)	1.00 [0.98–1.02]		1.00 [0.99–1.02]	0.659
Race				
White	Reference		Reference	
Black	1.30 [0.27–6.26]	0.742	1.26 [0.37–4.29]	0.714
Other	1.36 [0.27–6.61]	0.707	1.19 [0.35–4.07]	0.785
BMI (kg/m^2^)	1.00 [0.97–1.02]	0.899	1.00 [0.98–1.03]	0.925
Smoker				
Smoker	Reference		Reference	
Nonsmoker	1.37 [0.79–2.37]	0.267	1.08 [0.68–1.72]	0.737
Histology				
Squamous	Reference		Reference	
Adenocarcinoma	0.06 [0.01–0.64]	**0.020**	0.23 [0.03–1.90]	0.167
Adenosquamous	0.10 [0.01–1.14]	0.063	0.44 [0.05–4.07]	0.471
Stage				
IB2	Reference		Reference	
IIA	0.37 [0.12–1.21]	0.099	0.42 [0.18–1.00]	0.050
IIB	0.28 [0.07–1.14]	0.075	0.33 [0.12–0.85]	**0.022**
IIIA	0.69 [0.25–1.88]	0.463	0.40 [0.18–0.89]	**0.025**
IIIB	1.61 [0.47–5.55]	0.449	0.48 [0.14–1.62]	0.235
IVA	1.28 [0.52–3.12]	0.590	0.92 [0.46–1.84]	0.822
Treatment				
RT alone	Reference		Reference	
CRT	1.65 [0.63–4.31]	0.305	1.64 [0.74–3.60]	0.221
Circulating Neutrophil count	1.08 [0.99–1.17]	0.102	1.12 [1.03–1.21]	**0.006**
Circulating Lymphocyte count	0.67 [0.47–0.96]	**0.027**	0.79 [0.60–1.06]	0.113
Circulating Monocyte count	1.08 [0.99–1.17]	0.201	1.07 [0.49–2.35]	0.858
